# Supplementary material for: Adaptive thermal plasticity enhances sperm and egg performance in a model insect
Source: eLife. 2019 Oct 1;8:e49452. doi: 10.7554/eLife.49452 (PMC6773439; doi:10.7554/eLife.49452)
Supplement: Supplementary file 1. [file elife-49452-supp1.docx]

SUPPLEMENTARY TABLE 1

| **Offspring count (conditional model; N= 1080)** | | | | | | |
| --- | --- | --- | --- | --- | --- | --- |
| **Fixed/*Random* effects** | **coef** | **s.e. (coef)** | **z** | **p** | **Var** | **SD** |
| Intercept | –12.96 | 8.88 | –1.45 | 0.14 | - | - |
| Treatment (centered) | 6.30 | 4.95 | 1.27 | 0.20 | - | - |
| Regime (centered) | –46.59 | 4.94 | –9.42 | **0.0001** | - | - |
| Block (scaled) | -68.13 | 6.49 | –10.48 | **0.0001** | - | - |
| Treatment (centered):Regime (centered) | 44.71 | 10.01 | 4.46 | **0.0001** | - | - |
| *Blocks (as factor)* | - | - | - | - | 4.68 | 2.16 |
| *Male ID* | - | - | - | - | 2504.83 | 50.04 |
| *Residual* | - | - | - | - | 830.80 | 28.82 |

| **Offspring count (zero-inflation model)** | | | | | | |
| --- | --- | --- | --- | --- | --- | --- |
| **Fixed effects** | **coef** | **s.e. (coef)** | **z** | **p** | **Var** | **SD** |
| Intercept | 3.19 | 0.27 | 11.56 | **0.0001** | - | - |
| Treatment (centered) | 0.11 | 0.26 | 0.41 | 0.67 | - | - |
| Regime (centered) | 2.10 | 0.55 | 3.80 | **0.0001** | - | - |
| Block (scaled) | 4.72 | 0.34 | 13.51 | **0.0001** | - | - |
| Treatment (centered):Regime (centered) | –2.88 | 0.53 | –5.37 | **0.0001** | - | - |
| Regime (centered):Block (scaled) | 1.51 | 0.69 | 2.16 | **0.03** | - | - |
