# Supplementary material for: Adaptive thermal plasticity enhances sperm and egg performance in a model insect
Source: eLife. 2019 Oct 1;8:e49452. doi: 10.7554/eLife.49452 (PMC6773439; doi:10.7554/eLife.49452)
Supplement: Supplementary file 2. — Models were run using glmmTMB and were sorted along ascending AIC values. All conditional models additionally included random intercepts for ten-day blocks as well as random intercepts and random slopes for individual males. [file elife-49452-supp2.docx]

SUPPLEMENTARY TABLE 2

| conditional model | | | | | | | | | zero-inflation model | | | | | | | | |
| --- | --- | --- | --- | --- | --- | --- | --- | --- | --- | --- | --- | --- | --- | --- | --- | --- | --- |
|  | Intercept | Treatment | Regime | Block | Treatment : Regime | Treatment : Block | Regime : Block | Treatment : Regime : Block | Treatment | Regime | Block | Treatment : Regime | Treatment : Block | Regime : Block | Treatment : Regime : Block | df | AIC |
| Model1 | x | x | x | x | x |  |  | x | x | x | x | x | x |  | x | 16 | 3792.5 |
| Model2 | x | x | x | x | x | x |  | x | x | x | x | x | x |  | x | 17 | 3793.8 |
| Model3 | x | x | x | x | x | x | x | x | x | x | x | x | x |  | x | 19 | 3793.8 |
| Model4 | x | x | x | x | x | x | x | x | x | x | x | x | x | x |  | 20 | 3795.8 |
| Model5 | x | x | x | x | x | x | x |  | x | x | x | x | x | x |  | 19 | 3796.5 |
| Model6 | x | x | x | x | x |  | x | x | x | x | x | x | x | x | x | 19 | 3796.6 |
| Model7 | x | x | x | x | x | x |  | x | x | x | x | x |  |  | x | 16 | 3796.9 |
| Model8 | x | x | x | x | x | x |  | x | x | x | x | x | x | x | x | 19 | 3797.6 |
| Model9 | x | x | x | x | x | x | x | x | x | x | x | x | x | x | x | 21 | 3797.6 |
| Model10 | x | x | x | x | x |  | x | x | x | x | x | x |  | x | x | 17 | 3797.9 |
| Model11 | x | x | x | x | x | x | x |  | x | x | x | x | x | x | x | 20 | 3798.2 |
| Model12 | x | x | x | x | x | x | x | x | x | x | x | x |  | x | x | 19 | 3798.9 |
| Model13 | x | x | x | x |  |  |  | x | x | x | x | x | x |  | x | 15 | 3809.2 |
| Model14 | x | x | x | x |  | x |  | x | x | x | x | x | x |  | x | 16 | 3810.5 |
| Model15 | x | x | x | x |  |  |  | x | x | x | x | x |  |  | x | 14 | 3812.2 |
| Model16 | x | x | x | x |  |  |  | x | x | x | x | x | x | x | x | 17 | 3812.9 |
| Model17 | x | x | x | x |  | x | x | x | x | x | x | x | x | x | x | 19 | 3815.1 |
| Model18 | x | x | x | x | x | x | x | x | x | x | x |  | x | x | x | 19 | 3824.0 |
| Model19 | x | x | x | x | x | x |  | x | x | x | x |  | x |  | x | 16 | 3824.0 |
| Model 20 | x | x | x | x | x | x |  | x | x | x | x |  |  |  |  | 15 | 3828.4 |
| Model 21 | x | x | x | x | x | x | x | x | x | x | x |  |  |  |  | 17 | 3828.5 |
| Model 22 | x | x | x | x |  | x |  | x | x | x | x |  | x |  | x | 15 | 3840.8 |
| Model 23 | x | x | x | x |  | x | x | x | x | x | x |  | x | x | x | 17 | 3841.5 |
| Model 24 | x | x | x | x |  |  |  |  | x | x | x |  |  |  |  | 13 | 3843.9 |
| Model 25 | x | x | x | x | x |  |  | x | x | x | x | x |  |  | x | Did not converge | |
