# Supplementary material for: Adaptive thermal plasticity enhances sperm and egg performance in a model insect
Source: eLife. 2019 Oct 1;8:e49452. doi: 10.7554/eLife.49452 (PMC6773439; doi:10.7554/eLife.49452)
Supplement: Transparent reporting form [file elife-49452-transrepform.docx]

***eLife’s* transparent reporting form**

We encourage authors to provide detailed information *within their submission* to facilitate the interpretation and replication of experiments. Authors can upload supporting documentation to indicate the use of appropriate reporting guidelines for health-related research (see [EQUATOR Network](http://www.equator-network.org/%20)), life science research (see the [BioSharing Information Resource](https://biosharing.org/)), or the [ARRIVE guidelines](http://www.plosbiology.org/article/info:doi/10.1371/journal.pbio.1000412) for reporting work involving animal research. Where applicable, authors should refer to any relevant reporting standards documents in this form.

If you have any questions, please consult our Journal Policies and/or contact us: [editorial@elifesciences.org](mailto:editorial@elifesciences.org).

**Sample-size estimation**

- You should state whether an appropriate sample size was computed when the study was being designed
- You should state the statistical method of sample size computation and any required assumptions
- If no explicit power analysis was used, you should describe how you decided what sample (replicate) size (number) to use

Please outline where this information can be found within the submission (e.g., sections or figure legends), or explain why this information doesn’t apply to your submission:

Statistical tests were not conducted in advance of the experiments to plan sample sizes as the effect sizes were unknown before the research had been conducted. Balanced and reciprocal experiments were designed for execution within the study to minimise any confounding effects. Experimental methods, data analyses, replication levels, variance and sample sizes are reported in detail through the Methods and in Table 1 and 2.

Although the study used laboratory insects, the stored product pest Tribolium castaneum, not vertebrates or higher invertebrates, the research was approved within the ARRIVE guidelines for the use of animals in research and, as stated in the Methods section (Line 422): ‘*Approval for the research was granted by UEA's Animal Welfare and Ethical Review Board.*’

**Replicates**

- You should report how often each experiment was performed
- You should include a definition of biological versus technical replication
- The data obtained should be provided and sufficient information should be provided to indicate the number of independent biological and/or technical replicates
- If you encountered any outliers, you should describe how these were handled
- Criteria for exclusion/inclusion of data should be clearly stated
- High-throughput sequence data should be uploaded before submission, with a private link for reviewers provided (these are available from both GEO and ArrayExpress)

Please outline where this information can be found within the submission (e.g., sections or figure legends), or explain why this information doesn’t apply to your submission:

Experimental methods, repeats, data analyses, replication levels, sample sizes and variance are reported in detail through the Methods and in Table 1 and 2 and Supplementary Table 1 and 2. All replication was biological. All data from each experimental replicate were included for analysis, and no data were excluded. We include the statement in the Statistical Methods section (Line 587): ‘*All data generated from the experiments described above were included for analysis, and all replication is biological*.’)

**Statistical reporting**

- Statistical analysis methods should be described and justified
- Raw data should be presented in figures whenever informative to do so (typically when N per group is less than 10)
- For each experiment, you should identify the statistical tests used, exact values of N, definitions of center, methods of multiple test correction, and dispersion and precision measures (e.g., mean, median, SD, SEM, confidence intervals; and, for the major substantive results, a measure of effect size (e.g., Pearson's r, Cohen's d)
- Report exact p-values wherever possible alongside the summary statistics and 95% confidence intervals. These should be reported for all key questions and not only when the p-value is less than 0.05.

Please outline where this information can be found within the submission (e.g., sections or figure legends), or explain why this information doesn’t apply to your submission:

All statistical methods are described in fine detail in the Methods (Statistical Methods subsection). Data analyses, replication levels, sample sizes, effect sizes, model summaries and assumptions, AIC values and statistical differences are reported in detail through the Methods and in Table 1 and 2 and Supplementary Files 1 and 2. Summary statistics and exact P values are reported throughout to three decimal places, unless below 0.001 in which case P was reported as <0.001. All means (and medians) are presented on boxplots with interquartile ranges and, where possible and informative, raw data values as jitters.

(For large datasets, or papers with a very large number of statistical tests, you may upload a single table file with tests, Ns, etc., with reference to sections in the manuscript.)

**Group allocation**

- Indicate how samples were allocated into experimental groups (in the case of clinical studies, please specify allocation to treatment method); if randomization was used, please also state if restricted randomization was applied
- Indicate if masking was used during group allocation, data collection and/or data analysis

Please outline where this information can be found within the submission (e.g., sections or figure legends), or explain why this information doesn’t apply to your submission:

Group allocation is described for every experiment through subsection of the Methods. Balanced and reciprocal experiments were designed for execution within the study to minimise confounding effects. Individuals were allocated to experimental treatment groups randomly.

**Additional data files (“source data”)**

- We encourage you to upload relevant additional data files, such as numerical data that are represented as a graph in a figure, or as a summary table
- Where provided, these should be in the most useful format, and they can be uploaded as “Source data” files linked to a main figure or table
- Include model definition files including the full list of parameters used
- Include code used for data analysis (e.g., R, MatLab)
- Avoid stating that data files are “available upon request”

Please indicate the figures or tables for which source data files have been provided:

Raw source data and R code for all analyses and every Figure (including within the Supplementary Information) will be uploaded to Dryad on acceptance. The source data file will contain sections to enable each analysis and figure to be linked to the relevant source data and R code.

We include the following **data accessibility statement** on our cover page: ‘*All data generated and analysed in this study, together with R codes, are openly provided as an associated source file in our Dryad Data Repository with the identifier: https://doi.org/TBC ON ACCEPTANCE.’*
